# Optimization of somatic embryogenesis protocol for *Hevea brasiliensis* clones RRIM 600 and REYAN 88-13

**DOI:** 10.1016/j.jgeb.2025.100640

**Published:** 2025-12-20

**Authors:** Florence Dessailly, Li Zhe, Florence Martin, Julie Petit-Briand, Pascal Montoro, Julie Leclercq

**Affiliations:** a CIRAD, UMR AGAP Institute, F-34398 Montpellier, France; b UMR AGAP Institute, Univ. Montpellier, CIRAD, INRAE, Institut Agro, F-34398 Montpellier, France; c Rubber Research Institute, Chinese Academy of Tropical Agricultural Sciences/Key Laboratory for Biology and Genetic Resources Utilization of Rubber Tree, Ministry of Agriculture/State Rubber Tree Breeding Center, Danzhou, Hainan 571737, China

**Keywords:** Rubber tree, Secondary somatic embryogenesis, *Agrobacterium tumefaciens*, Cocultivation, Genetic transformation, Rubber

## Abstract

The challenges for the development of rubber cultivation are so agronomically significant in the face of climate change that it is crucial to introduce biotechnology into the clonal propagation of rubber trees. A protocol was developed on rubber clone PB 260. This protocol was used for two other clones, RRIM 600 and REYAN 88-13, which exhibited highly contrasting response to callus growth and embryogenic capacity. Optimization of somatic embryogenesis for these two clones led us to successful conduct genetic transformation trials. We discussed the *in vitro* behaviour of the calli considering the origin of the embryogenic callus and the endogenous redox status.

## Highlights

• Optimization of somatic embryogenesis protocol revealed the predominant role of balances in growth regulators.
• This optimization should simultaneously control callus growth, tissue browning and embryogenic capacity of cells.
• An optimized protocol for somatic embryogenesis facilitates the production of transgenic lines.

## 1 Introduction

Tree breeding is challenging due to long generation cycles, late flowering and limited vegetative propagation^1^. Adaptability to climate change and multi-purpose usage puts intense selection pressure on cultivated trees^2^. Modern genetic improvement strategies integrate biotechnological methods such as in-vitro propagation, genetic transformation and marker-assisted selection^3^, ^4^, with genomics^5^ and phenomics^6^. In *Hevea*, breeding is hampered by issues such as low female fertility, seed viability^7^, graft incompatibility^8^ and loss of juvenility. To improve efficiency and stress resistance, advanced techniques are being integrated into conventional breeding programmes (https://rubberclones.cirad.fr/) (for review.^9^, ^10^, ^11^, ^12^, ^13^, ^14^).

**Fig. 1:**
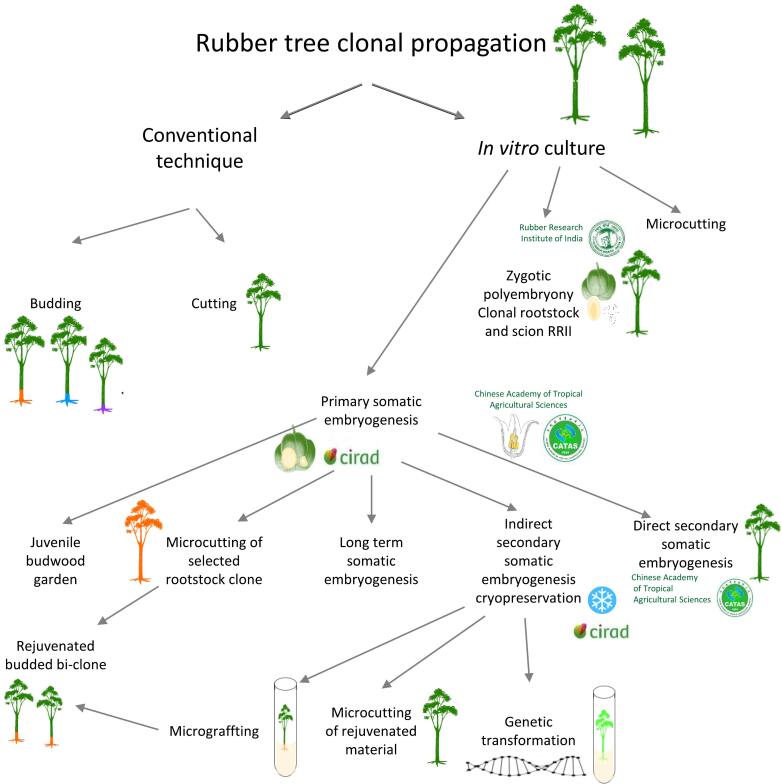
Integration of biotechnological *in vitro* culture approaches in the clonal propagation of rubber trees.

*Hevea* is highly heterozygous and strictly allogamous, leading to considerable heterogeneity in the first rubber plantations planted with seedlings. Since the emergence of grafted rubber tree clones, the homogeneity of these plantations has been improved, even though the rootstocks are still grown from seedlings, maintaining some phenotypic variability. Cutting has also some limitations in terms of multiplication rate and root anchoring in the ground^15^. In-vitro clonal propagation poses agronomical challenges in *Hevea*, requiring the preservation of juvenility to enable vegetative multiplication and conserve genetic diversity. This leads to the creation of various varietal types, the synthesis of which is presented in Fig. 1. In-vitro culture overcomes these limitations by producing clonal rootstocks, scions, and self-rooted plants, offering alternatives to sexual reproduction (Fig. 1). Commercially operational microcutting techniques provide homogeneous plants and disease resistance^16^, ^17^, ^18^, ^19^ (Fig. 1). Rubber cultivation would benefit from all these innovations to enhance its agronomic performance.

Maintaining juvenility for clonal propagation is crucial. Somatic embryogenesis (SE) makes it possible in *Hevea* to rejuvenate plant material suitable for microcutting and rejuvenated grafted plants^15^, ^20^, ^21^. Primary somatic embryogenesis has been initiated from various tissues, including anther, root, and internal tegument of immature fruits^15^, ^20^, ^22^, ^23^, ^24^, ^25^, ^26^, ^27^ but it does not yet provide the embryogenic capacity needed for mass propagation. In this process, somatic embryos are produced by a primary callus. As this callus turns brown during embryo induction, the number of embryos is very low. Therefore, two secondary somatic embryogenesis processes have been developed for the long-term and large-scale production of somatic embryos (Fig. 1). Direct secondary somatic embryogenesis was developed by CATAS in China^28^. The primary callus regenerates primary embryos, which are used as explants to regenerate secondary embryos without callus production. At CIRAD, an indirect secondary somatic embryogenesis process has been developed for clone PB 260. It consists of initiating a compact primary callus with friable aggregates, which are used to establish secondary embryogenic and friable callus lines, from which embryos are produced. Embryogenic cell proliferation is controlled by a balance of growth regulators^29^, ^30^, while the callus friability is induced by a high concentration of calcium. This process is time-limited through cryopreservation of friable callus to mitigate somaclonal mutations arising from extended in-vitro culture^31^. This process yields cryopreserved regenerating callus stocks, which are available for transgenesis and plant production using RITA® (temporary immersion system, CIRAD, France)^32^. The process was subsequently applied at CIRAD to the clones PB 217 and RRIM 703^31^. In *Hevea*, the micropropagation technique from in-vitro plants obtained through indirect secondary somatic embryogenesis has been developed with the aim of producing clonal rootstocks (Fig. 1). Similarly, a micrografting technique has also been developed^33^, enabling the creation of aerial and root bi-clones (Fig. 1)^15^, ^24^. Furthermore, in-vitro plants derived from indirect secondary somatic embryogenesis can supply juvenile scions for planting.

Due to the expertise gained at CIRAD in somatic embryogenesis techniques, it has been observed that adapting established protocols for clone PB 260 is not easily transferable to other clones of agronomically valuable interest, which may vary in their origins and responsiveness. Clone RRIM 600 was developed in Malaysia and is the clone the most planted especially in Thailand, the leading rubber producing country. This clone is well adapted to cold in China and drought stress in India, Thailand and even in Indonesia. Clone REYAN 88-13 was one of the first clones created in Hainan province in China and well-adapted to the wind conditions. These two clones show also very contrasting in-vitro behaviours. Callus lines from clone RRIM 600 are derived from indirect secondary somatic embryogenesis and react strongly to in-vitro culture stress. These lines have a poor multiplication rate due to callus browning and excessive uncontrolled embryo production. Conversely, lines from clone REYAN 88-13 are originated from secondary embryos from anther culture without primary callogenesis. These callus lines have a high multiplication rate and low embryo production capacity.

Here, we describe step-by-step the points for optimizing the protocol for clone PB 260 to maximize embryo production in terms of quality and quantity. Culture containers, culture times and modification of growth regulators balances were crucial adjustments for successful development of protocols optimized for the RRIM 600 and REYAN 88-13 clones, respectively. We also discussed clonal differences that may explain these contrasting in-vitro behaviours.

## 2 Material et methods

### 2.1 Standard basal medium for the PB 260 clone

The basic composition of the media (MM) is as follows: macro-elements (NH_4_NO_3_: 200 mM, KNO_3_: 200 mM, MgSO_4_·7H_2_O: 30 mM, NaH_2_PO_4_·H_2_O: 20 mM), micro-elements (H_3_BO_3_: 150.08 µM, MnSO_4_·H_2_O: 100 µM, ZnSO_4_·7H_2_O: 40 µM, CuSO_4_·5H_2_O: 1.48 µM, Na_2_MoO_4_·2H_2_O: 0.99 µM, KI: 5 µM, CoCl_2_·6H_2_O: 1.01 µM), vitamins (inositol: 300 nM, nicotinic acid: 20 nM, pyridoxine-HCl: 3 nM, thiamine-HCl:2 nM, biotine: 0.2 nM, D- calcium pantothenate: 1 nM, ascorbic acid: 1 nM, choline chloride: 1 nM, L-cysteine-HCl: 60 nM, glycine: 5 nM, riboflavin: 1 nM), 9 mM CaCl_2_, 30 µM AgNO_3_, 234 mM sucrose and 2.3 g.L^−1^ Phytagel^20^, ^34^. To adapt the culture media to RRIM 600 and REYAN 88-13 calli, several plant growth regulators concentrations were tested for benzylaminopurine (BAP from 0 up to 4.44 µM), 3,4-dichlorophenoxyacetic acid (3,4-D from 0 up to 4.52 µM) and abscisic acid (ABA from 0 up to 4 µM). The pH of all media was adjusted to 5.8 prior to autoclaving. Cultures are grown in the dark at 27 °C.

### 2.2 Obtaining friable callus for the PB 260, RRIM 600 and REYAN 88-13 clones and their cryopreservation (Fig. 2 steps 1 to 4)

**Fig. 2:**
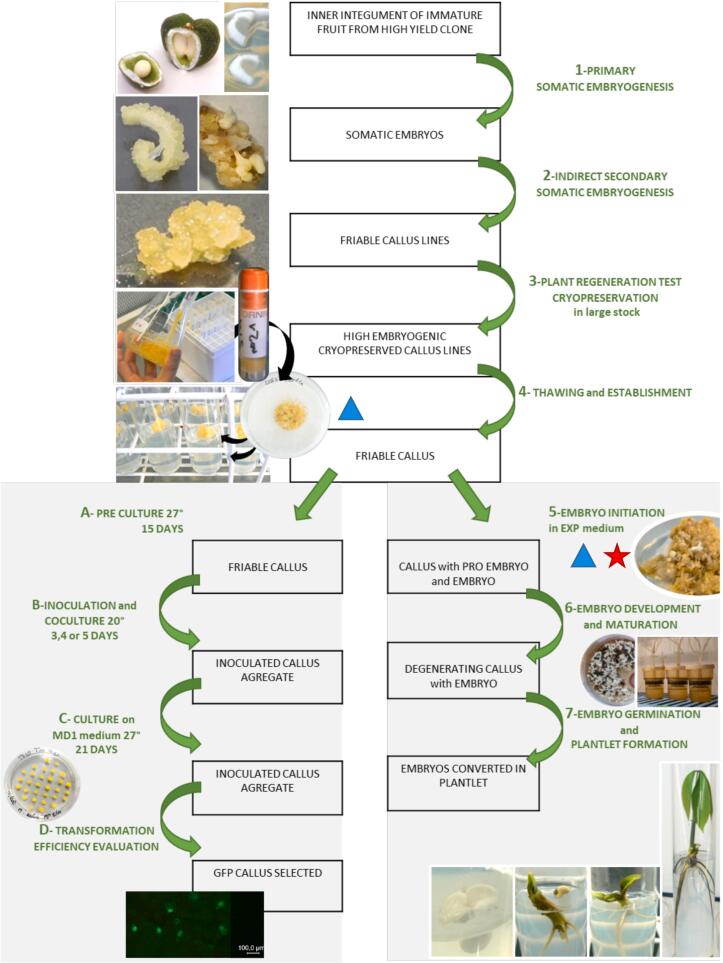
Description of thesomatic embryogenesis process established for the PB 260 clone. The modified protocol steps are represented for the RRIM 600 clone by a blue triangle and for the REYAN 88-13 clone by a red star. (For interpretation of the references to colour in this figure legend, the reader is referred to the web version of this article.)

This process is divided into three parts (Fig. 2 steps 1 to 4). The first part aims to obtain friable callus. To produce a friable callus for clone PB 260 and RRIM 600, the starting explant is a slice of the inner integument (maternal part) of immature fruits of the selected genotype. Primary embryo formation is induced after several cycles of culture on the MH1, MH2 and MH3 media described by Lardet et al.^31^. Once a primary embryo is obtained, it is then cultured on INF medium to produce friable callus (indirect secondary embryogenesis, Table 1). The callus friability is induced by a balance of growth regulators and calcium that controlled the multiplication of embryogenic cells and the secretion of mucilage^35^, ^36^. Fifteen calli (the initial number of aggregates) were multiplied through three subcultures on control medium and six treatments with varying concentrations of auxins (3,4-D, BAP) and ABA. Two containers were tested in a preliminary experiment, (data not shown). The decision was made to use glass tube for callus propagation in the case of REYAN 88-13 and Petri dish plates for RRIM 600. At the end of the third subculture, the following parameters of the callus were monitored: browning appearance (regular callus: 0 % browning, brown callus: less than 50 % browning and very brown callus: more than 50 % browning), spontaneous embryo production (two categories: callus without any embryos and callus bearing embryos), callus proliferation level (three categories according to their proliferation rate: no proliferation, by 1.5, by 2 and by more than 2), regenerative capacity in embryos (number of well-shaped embryos / g of callus), and plant regeneration ability (number of plant/ g of callus).

**Table 1:** Exhaustive summary of the changes made in the culture media (in white) compared to the standard protocol developed for clone PB 260 (in grey).

Once stabilized, the callus line is cryopreserved according to a protocol described in Engelmann et al. 1997^37^, involving two successive steps: a cryoprotective treatment and then a freezing step. The embryogenic and regeneration capacities are then tested as described below. Traceability is ensured through a Microsoft Access database called BIOTECKVA^38^. For clone REYAN 88-13, the initial explant was obtained from an anther culture at CATAS. Dr Li Zhe provided the calli from secondary embryogenesis.

### 2.3 Medium for regeneration for the PB 260, RRIM 600 and REYAN 88 clones (Fig. 2 steps 5 to 7)

In the second part (Fig. 2 steps 5 to 7), after thawing and amplification of a chosen callus line, plant production can be initiated. This process was also described by Lardet et al. (2009)^30^. The callus can be induced for regeneration after thawing cryopreserved callus and amplification of a selected line in glass tubes with ENT medium. At this stage, no changes were made to the medium for the culture of the callus from REYAN 88-13 clone. By contrast, due to the behaviour of the RRIM 600 callus, changes were made to the culture container (Petri dish plate instead of glass tubes), time of subculture (21 days instead of 14 days) and to the ENT medium with a different concentration of growth factors with the removal of ABA and the increase in 3,4-D and BAP concentrations (Table 1).

Regeneration is initiated on an EXP medium (Table 1). For the RRIM 600 clone, a preEXP culture step was introduced to acclimatise the callus to lower growth factor concentrations in the EXP medium. While no changes in growth regulators concentrations were required for the clone RRIM 600 in the EXP medium, the growth regulators concentrations in EXP medium for the REYAN 88-13 clone was modified. The improvement of the protocol proposed for REYAN 88-13 clone concerns the initiation of embryogenesis (Fig. 2, Step 5). ABA concentrations ranging from 0.05 to 4 µM were tested in the absence of other growth regulators (3,4-D and BAP). After induction of embryogenesis, the callus is then matured in RITA® using DEV1 and DEV2 media. After maturation in RITA under standard conditions (Fig. 2, Step 6), the produced embryos were classified into two categories according to their quality. Well-shaped embryos exhibit two essential characteristics, namely a marked polarization of the root and shoot poles, and more or less a bilateral symmetry of the cotyledons. In contrast, malformed embryos have aberrant embryonic structures, some lacking embryonic bodies, others being deformed, spherical, with fused structures. The embryos are harvested and isolated on solid DEV3 development medium, which allows for the conversion of embryos into plants (Table 1^,^ Fig. 2, Step 7).

### 2.4 Genetic transformation of friable calli from PB 260, REYAN 88-13, and RRIM 600 clones (Fig. 2 steps A to D)

Alternatively, a third step of genetic transformation can be inserted before plant production (Fig. 2 steps A to C). In this case, after thawing and establishment of the line, the callus is conditioned before coculture with *Agrobacterium tumefaciens*. The selection of transformation events is done using the GFP reporter gene^38^. For the transformation experiments, the calli from three clones were thawed and callus lines established^23^, ^38^. To test the transformability of the clones, we considered improvements in callus proliferation management. Calli were cultured in tubes for PB 260 and REYAN 88-13, and in Petri dish plates for clone RRIM 600. A preculture cycle in a calcium-free medium (MP, corresponding to the ENT medium, for REYAN 88-13 and PB 260, and ENT-0Ca for RRIM 600) for 13 days in tubes for PB 260 and REYAN 88-13, or 16 days in Petri dishes for RRIM 600 was added. The actual transformation is achieved by coculturing for 2 to 4 days with *Agrobacterium tumefaciens* containing a binary vector carrying the GFP reporter gene^38^. Agrobacteria were grown in liquid Lysogeny Broth medium (Duchefa, Haarlem, The Netherlands) supplemented with 50 mg.L^−1^ kanamycin and 100 µM acetosyringone at 28 °C until OD_600nm_ = 0.6. The pellet, obtained after centrifugation at 1,000 g for 10 min, was dissolved to OD_600nm_ = 0.06 in liquid MM medium from which Fe-EDTA, CaCl_2_ and growth regulators were eliminated and to which 100 µM acetosyringone^23^. The inoculated callus (Fig. 2, Step B) is then transferred into aggregates on a decontamination medium MD1 (Fig. 2, Step C), a ENT medium containing 500 mg. L^−1^ ^23^ (Table 1). The GFP screening was performed under a fluorescence stereomicroscope (MZ FLIII, Leica Microsystems, Wetzlar, Germany) using the GFP2 filter (480 nm excitation filter/ 510 nm barrier filter). The number of transgenic units was recorded on GFP fluorescent calli after a 21-day cultivation on MD1 medium, where several coculture durations (2,3 and 4 days) were tested (Fig. 2, Step D). The transgenic units were calculated from the spots and clusters exhibiting GFP observed on 50 aggregates.

### 2.5 Statistical analyses

All experiments are performed with biological replications. By using XLSTAT (Addinsoft, Paris, France), and after performing a normality test, an ANOVA analysis followed by a two-tailed Student Neuman-Keuls test were used in all statistical analyses (p < 0.05). Error bars correspond to the standard errors calculated as the as σ/√ (number of replicates).

## 3 Results

### 3.1 Protocol adaptations for somatic embryogenesis in the RRIM 600 clone

**Fig. 3:**
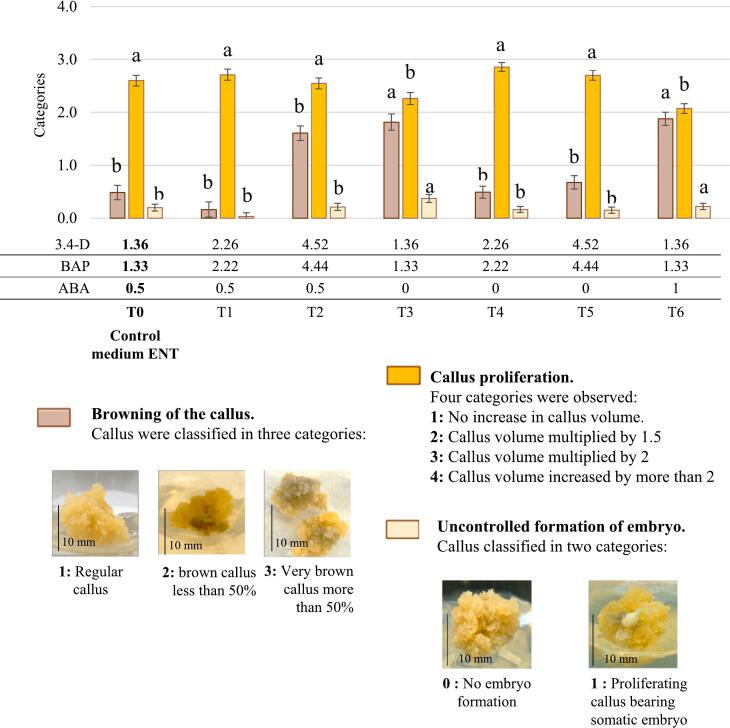
Illustrations of the consequences of changes in culture media on somatic embryogenesis for the RRIM 600 clone. This experiment was carried out with 15 initial calli for each treatment. After the third subculture, quality of callus and presence of embryo in proliferating callus were observed. Browning appearance: Three categories are observed category 1) Regular callus: 0% browning, category 2) Brown callus: less than 50% browning and category 3) Very brown callus: more than 50% browning. Callus proliferation: we classified callus in four categories according to their proliferation rate: category 1) no increase in callus volume, category 2) by 1.5, category 3) by 2 and category 4) by more than 2.

**Fig. 4:**
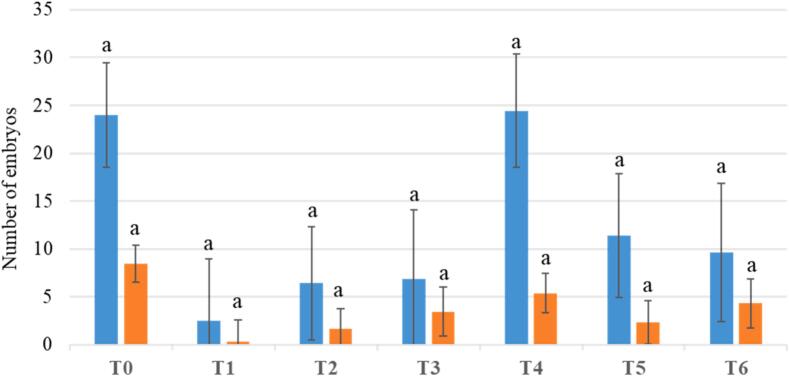
Number of embryos (blue bar) and plantlets (orange bar) per gram of embryogenic callus. The regeneration of embryos and plantlets correspond to step 5 to step 7 in Fig. 2. (For interpretation of the references to colour in this figure legend, the reader is referred to the web version of this article.)

The optimizations described below have favored the callus proliferation phase. Using the standard protocol, RRIM 600 callus turns brown and generates spontaneous embryonic forms during the callus multiplication cycles, indicating uncontrolled induction of the embryogenesis process. This phenomenon is irreversible, and callus degenerates. Protocol optimization involved boosting cell multiplication by manipulating hormonal balances (3,4-D, BAP, and ABA). Based on Fig. 3, an increase in auxin quantity appears to promote healthy callus proliferation. Treatments T1, T4, and T5 are particularly effective, with a rate of over 80 % healthy callus and fewer than 15 % of aggregates bearing spontaneous embryos (Fig. 3). Despite the same initial quantity of callus distributed in 15 tubes during the first culture cycle, the production of callus after 3 culture cycles is higher for treatments T1 (4.2 g), T4 (5 g), and T5 (4.2 g), surpassing callus production for the control treatment, with T4 having the highest advantage (Fig. 3). The callus harvested after 3 culture cycles for each treatment was then evaluated according to the standard regeneration protocol (Fig. 2, Steps 5–7). The control treatment allows for the highest production of embryos (37) and seedlings (14). Treatment T4 yields a similar embryo production to the control treatment (36) but with fewer plantlet conversions (8). Treatments T1 and T5, although effective in reducing browning and controlling spontaneous embryogenesis, do not result in satisfactory embryo production and, consequently, plantlet production (Fig. 4). The proposed optimization for the RRIM 600 clone involves cultivation on the T4 proliferation medium. The transition from proliferation to regeneration is facilitated by the addition of an intermediate culture step, allowing for a more gradual decrease in hormonal concentration (pre-EXP in Table 1). Regeneration can then proceed following the standard protocol (T0 treatment). Since the callus struggles with proliferation, the container has also been changed to Petri dishes, which are more conducive to the culture of small aggregates, allowing for a culture duration of 21 days instead of 14 for tube culture (data not shown).

### 3.2 Protocol adaptations for the REYAN 88-13 rubber clone

**Fig. 5:**
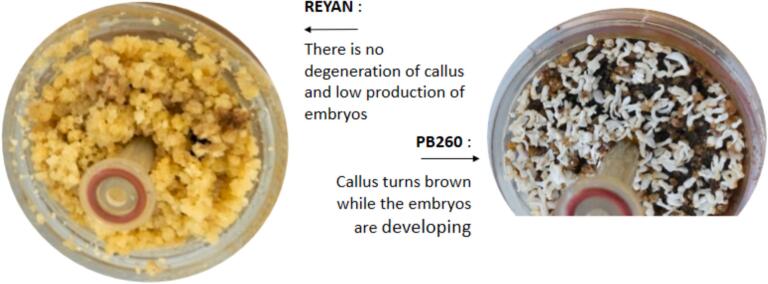
Comparison of callus evolution of clones PB 260 and REYAN 88-13 at the end of embryo production in RITA® (Step 5 on Fig. 2).

**Fig. 6:**
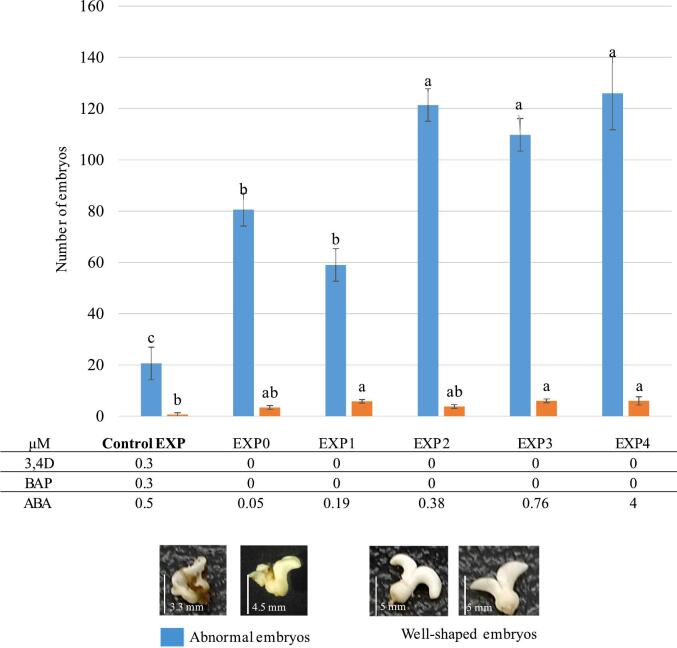
Illustrations of the consequences of changes in culture media on somatic embryogenesis for the REYAN 88-13 clone. Values with the same letter are not significantly different at the 0.05 probability level.

Throughout the somatic embryogenesis process, the callus of REYAN 88-13 clone behaves quite differently compared to the one of PB 260 clone. Indeed, for the PB 260 clone, embryo production is accompanied by browning of the callus (Fig. 5). On the other hand, the callus of the REYAN 88-13 clone proliferates greatly but does not respond to reference media for embryogenic expression. The callus remains yellow and produces very few embryos, which are also poorly formed (Fig. 6).

For the REYAN 88-13 clone, the suppression of auxins is accompanied by a significant increase in the number of well-shaped embryos (Fig. 6). Increasing the ABA concentration also leads to a significant increase in abnormal embryo production (Fig. 6). In this experiment, the EXP1 treatment shows the best ratio of well-shaped to unwell-shaped embryos and limits the competition of malformed embryos in the RITA®.

### 3.3 Attempt at genetic transformation of calli using optimized culture conditions

**Fig. 7:**
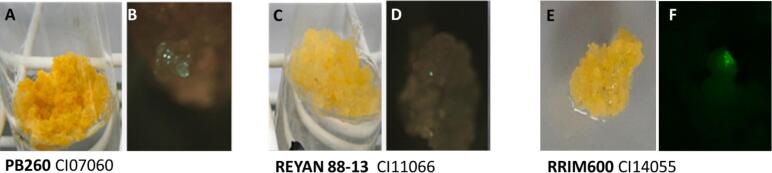
Transformation efficiency, recorded by GFP fluorescence on small aggregates 14 days after cocultivation of friable calli of clone PB 260 (**A**. before coculture, **B**. GFP visualisation), REYAN 88-13 (**C**. before coculture, **D**. GFP visualisation) and RRIM 600 (**E**. before coculture, **F**. GFP visualisation).

Mastering indirect secondary embryogenesis opens possibilities for genetic transformation, as shown in Fig. 1^22^, ^35^, ^38^, ^39^, ^40^. Callus transformation allows the isolation of fully GFP lines, eliminating the risk of chimeric plants^38^. GFP expression is observed after 21 days of culture (Fig. 7 A-F). In Table 2, fluorescence is visible in calli cocultured with *Agrobacterium* for 2 days for RRIM 600, 3 days for REYAN 88-13, and 4 days for PB 260. The number of transformation events increases with coculture duration for all three clones. However, the balance between transformation efficiency and callus viability must be considered, with *Agrobacterium* infection controlled by the addition of the bacteriostatic agent ticarcillin in the culture medium MD1.

**Table 2:** Transformation efficiency, expressed as the number of transformation units per gram of fresh weight, measured by GFP fluorescence on small aggregates 14 days after cocultivation of friable calli of clone PB 260, REYAN 88-13, and RRIM 600 with *Agrobacterium tumefaciens*. Values with the same letter are not significantly different at the 0.05 probability level.

Clone	Repetition number	Coculture duration	Normalisation GFP Nb events
PB260	5	2	0,000^b^
PB260	5	3	0,000^b^
PB260	5	4	7,181 ^a^
REYAN 88-13	5	2	0,000^c^
REYAN 88-13	5	3	2,603^b^
REYAN 88-13	5	4	5,102 ^a^
RRIM600	6	2	1,299^b^
RRIM600	6	3	1,671^b^
RRIM600	6	4	3,112 ^a^

## 4 Discussion

### 4.1 Difficulties in the development of somatic embryogenesis in multiple *Hevea* clones

Many attempts to vegetatively propagate elite rubber clones by somatic embryogenesis were described (for review^15^). In collaboration with CIRAD, procedures for primary somatic embryogenesis were established by RRIT in Thailand for clone BPM 24 and Michelin's CPN laboratory for clones BPM 24, PR 107, RRIM 600, PB 254, IRCA 109, IRCA 317, PB 260, RRIM 703, PB 217, and IRCA 41. Other research institutes (CATAS in China, RRIM in Malaysia, RRII in India, and IBRIEC in Indonesia) developed procedures using anther culture for clones Haiken 1, Haiken 2, GL1, Dafeng 95, REYAN 7-33-97, Wenchang 217, Yun Yan 77-2, RRII 105, and PR 300. However, the presence of laticifers in the callus inhibits somatic embryogenesis for genotypes PR 107, RRIM 600, REYAN 8-79, REYAN 7-33-97, and Haiken 2^41^, ^42^, ^43^. Primary embryogenesis led to a low-rate multiplication compared to long-term somatic embryogenesis using friable calli but with somaclonal variation^24^, ^34^. High embryogenic ability of friable calli from a primary embryo explant was obtained at CIRAD (secondary embryogenesis) for clone PB 260, and a cryopreservation step was introduced into the process to mitigate somaclonal variation occurrence and fix rapidly the callus lines^34^, ^40^. However, the adaptation of this protocol to other clones is difficult due to clonal characteristics. Only a few embryogenic callus lines were obtained for three clones PB 260, PB 217 and RRIM 703. This was due to clonal specificity for growth, callus friability, embryogenic potential, and tissue browning in response to in-vitro stress^30^, ^44^, ^45^. In this article, the mastery of indirect secondary embryogenesis for clones RRIM 600 and REYAN 88–15 was obtained thanks to growth control while maintaining the embryogenic capacity of calli. This work demonstrates the necessity of optimizing somatic embryogenesis protocols to each rubber tree clone and the predominant role of growth regulator balances. Three types of clones can be considered: low, medium and high growth. Testing more clones will enable us to determine whether the three protocols are suitable for them and promote the faster adaptation of friable embryogenic callus lines in future.

### 4.2 Control of growth, development and friability of calli by plant growth regulators and calcium application

Working with calli from three rubber clones exhibiting contrasting in-vitro culture responses, we demonstrated the difficulties to establish a single protocol for all clones. Indeed, protocol optimization needs to be done on a case-by-case basis, depending on the bottlenecks identified during somatic embryogenesis and plant regeneration, and potentially also during the genetic transformation steps.

For callus initiation, friability is generally obtained with a very high calcium content^35^, ^46^, ^47^. The clones RRIM 600 and REYAN 88-13 gave compact and friable calli with the calcium content of the standard protocol at culture initiation (12 mM). Only the friable callus could be multiplied until primary embryos were produced. The friable calli described in this article were obtained from explants derived from primary embryos. The friable calli from three rubber clones range from the least prolific (RRIM 600) to the most prolific (REYAN 88-13), from the least embryogenic (REYAN 88-13) to the most embryogenic (PB 260), but with uncontrolled embryogenesis. Secondary somatic embryogenesis requires further growth support to facilitate embryo formation. For clone RRIM 600, callus growth needs to be boosted by increasing plant growth regulators concentrations and reducing ABA concentration. Regeneration requires an intermediate conditioning step. Conversely, for clone REYAN 88-13, callus growth should be slowed down with the application of a high concentration of ABA. It is possible that this clone has an endogenous capacity to produce plant growth regulators. This may be related to the origin of the callus, which comes from secondary somatic embryogenesis conducted in China, likely requiring significant hormonal stimulation for the establishment of callus lines. Indeed, the improvement of the somatic embryogenesis in step 5 (Fig. 2) for the REYAN 88-13 clone may not be sufficient since it overreacts to growth regulators present from callus initiation (3,4-D and kinetin then BAP). It is possible that reducing concentrations during the proliferation phase may also be favourable for somatic embryogenesis but has not been tested.

The problem of callus browning has been resolved by regulating the proliferation of RRIM 600. When the callus begins to produce embryos, it turns brown. During multiplication, the callus must remain yellow. It was necessary to find the right culture conditions to maintain proliferation without spontaneous embryo production, which is associated with browning. A substantial gain in embryo morphogenesis was achieved by changing the carbon source of the embryo induction medium from sucrose to maltose^22^. This knowledge was also incorporated into the protocols for the clones studied in this paper. To be embryogenic, the callus must be in a sustained growth phase and rich in meristematic cells^32^, ^48^, ^49^. In this experiment, durable callus growth was achieved but the histological quality of the callus was not tested.

### 4.3 Implications of the redox state in the process of somatic embryogenesis in *Hevea brasiliensis*?

Callus tissue browning occurs when callus growth rate is low and under stressful culture conditions. For that reason, concentration in growth regulators should be adapted to favour callus growth^30^, ^49^. The addition of treatments controlling polyphenols, as well as inhibitor of ethylene perception was also successfully tested.^44^, ^50^ Interestingly, some authors could also controlled tissue browning by improving the water status of callus with specific environmental culture conditions^51^. In the step of induction of somatic embryogenesis, callus browning is observed due to the decrease in growth regulator concentration^20^.

Callus lines from clones presented in this study have contrasting growth rate and SE abilities, clone PB 260 have an intermediate growth rate and the highest SE production. Clone RRIM 600 requires high growth regulator concentration to support its low callus growth and prevent its uncontrolled production of somatic embryos. Conversely, the callus line of clone REYAN 88-13 is highly prolific and stand yellow even at low growth regulator concentration such as in the EXP medium. This led to a low embryogenic expression on these yellow callus tissues. It exhibits characteristics of callus lines overexpressing *HbCuZnSOD*^52^. These callus lines did not undergo the expected browning process in RITA® and produced a significantly lower number of embryos than anticipated. This suggests that ROS-scavenging enzymes may protect the callus from oxidative stress in clone REYAN 88-13.

### 4.4 Suitability for genetic transformation

Several clones are widely propagated by primary somatic embryogenesis in France, India and Malaysia and by direct secondary SE in China, and indirect secondary SE in France (for review^15^). Genetic modification studies have also been reported in these countries^52^, ^53^, ^54^, ^55^, ^56^, ^57^, ^58^. In France, the callus line CI07060 of clone PB 260 derived from indirect secondary embryogenesis, has been widely and successfully used for genetic transformation^23^, ^38^, ^52^, ^58^, ^59^. Controlling embryo formation from a fully GFP callus has enabled the production of thousands of homogeneous and non-chimeric transgenic plants^38^. This robust and efficient protocol was used for functional analyses of several candidate genes involved in ROS-scavenging systems and ethylene signalling pathway^52^, ^58^, ^59^.

However, although these transgenic plants could be studied only at the juvenile stage in confined greenhouse according to the GMO regulation in EU, the development of this methodology is increasingly important for functional studies and genome editing. In this paper, we reported the feasibility of genetic transformation for two additional clones, RRIM 600 and REYAN 88-13. The use of genome editing with CRISPR/Cas9 in protoplasts has also been reported^60^, ^61^ and the authors argue that this could increase latex yield and produce early-flowering rubber tree plants, thereby shortening the breeding cycle.

The embryogenic friable callus transformation used in this study has the advantage of reducing the risk of producing chimeric plants. To date, all transgenic lines used in functional studies at CIRAD generated uniformly transgenic plants.

## 5 Conclusions

A somatic embryogenesis protocol previously established for clone PB 260 was optimized in this study for two commercial rubber clones, RRIM 600 and REYAN 88-13. This study has shown the essential role of the balance of growth regulator concentrations. It has been shown that the culture conditions must allow the control of callus growth and friability, as well as overcoming tissue browning. Histological studies were essential in determining the culture conditions favourable to the development of embryogenic cells. *Agrobacterium tumefaciens*-mediated transformation was successfully attempted on the newly established embryogenic friable callus lines. The knowledge gained from this study should make it easier to optimize the somatic embryogenesis protocol for other commercial clones. This work will allow evaluating in-vitro plants in the field with a view to mass propagation for rubber plantations. Finally, embryogenic callus lines could be used for functional analyses of candidate genes.

## Funding and declaration of interest statement

This work for Dr LI Zhe was supported by the National International Scientific and Technological Cooperation Project (2008DFA32020) from Chinese Ministry of Science and Technology (MOST): « Studies on friable embryogenic callus long-term subculture, cryopreservation and plant regeneration in *Hevea brasiliensis »*; the “948” Project from Chinese Ministry of Agriculture (2010-S7): « Friable embryogenic callus long-term subculture and overexpressing *Lec1* gene for promoting somatic embryogenesis and plant regeneration in *Hevea brasiliensis »;* and the EGIDE grant n°718238D. All other authors did not receive any specific grant from funding agencies in the public, commercial, or not-for-profit sectors.

## CRediT authorship contribution statement

**Florence Dessailly:** Writing – original draft, Methodology, Formal analysis, Conceptualization. **Li Zhe:** Methodology, Formal analysis, Conceptualization, Project administration, Funding acquisition. **Florence Martin:** Validation. **Julie Petit-Briand:** Validation. **Pascal Montoro:** Writing – review & editing, Writing – original draft, Supervision, Project administration, Funding acquisition, Formal analysis, Conceptualization. **Julie Leclercq:** Writing – review & editing, Writing – original draft, Project administration.

## Glossary

SE: somatic embryogenesis
MM: basal medium
BAP: benzylaminopurine
3,4-D: 3,4-dichlorophenoxyacetic acid
ABA: abscisic acid
MH1, MH2, and MH3 media: callogenesis induction medium
INF medium: friability induction medium
EXP medium: embryo formation medium
RITA: temporary immersion system
DEV1, DEV2 and DEV3 media: embryo development media
MP: preculture medium
MD1: decontamination medium 1
GFP: green fluorescent protein
